# A nutritional perspective on cellular rejuvenation

**DOI:** 10.18632/oncotarget.4326

**Published:** 2015-06-01

**Authors:** Patricia Huebbe, Anke Schloesser, Gerald Rimbach

**Affiliations:** University of Kiel, Kiel, Germany

Restriction of dietary energy intake is widely accepted as the most powerful and robust intervention to extend lifespan and decelerate aging processes. Throughout the literature, the terms ‘calorie restriction’ and ‘dietary restriction’ are used synonymously, although substantial differences exist between them. Calorie restriction (CR) is achieved by decreasing the energy density of the diet by modifying dietary composition, whereas dietary restriction (DR) is accomplished by simply reducing the amount of allocated food, making DR much less laborious than CR. In addition, mice maintained under DR periodically undergo fasting because they tend to eat the daily provided food shortly after feeding, whereas mice fed *ad libitum* have a continuous supply of excess calories (this would be the case if CR is accomplished by decreasing the energy density of food, e.g., through the addition of non-digestible cellulose). However, evidence suggests that the periods of fasting that occur under standard DR regimens are crucial to the lifespan-extending effects, and the reduction in energy density alone fails to extend lifespan, at least in mice and flies (references found in Simpson et al. [1]). The underlying reasons for this discrepancy may include the deactivation of nutrient-sensing anabolic pathways during fasting. The most likely candidate and suitable indicator of lifespan extension by this intervention is the inhibition of mTOR activity [2].

The mechanistic target of rapamycin (mTOR) is a growth-promoting signaling pathway that is activated by circulating branched amino acids, insulin and other growth factors and drives cellular protein synthesis and growth. However, mTOR counteracts cellular cleansing and rejuvenation by inhibiting autophagy and promotes metabolic derangement, such as insulin receptor resistance. Mutant models with defective mTOR activity show that increased lifespans and are no longer responsive to lifespan extension by DR (references found in Cuervo [3]), whereas mTOR hyperactivation triggered by constantly circulating nutrients leads to cellular senescence and aging. In this context, ad libitum feeding or overeating can promote an aging phenotype. Mouse strains prone to diet-induced obesity (DIO) such as C57BL6 mice would therefore exhibit accelerated aging, especially when fed an energy-dense diet. This is of particular interest because the use of ad libitum (AL) fed controls has been criticized elsewhere on the grounds that AL animals were sedentary, obese and prone to premature death. It was questioned whether overweight AL animals could be suitable controls for the investigation of longevity. However, because AL feeding promotes obesity, and obesity favors age-related pathologies, a decrease in adverse AL effects would therefore prove its anti-aging potential. Interestingly, variance in the magnitude of DR-related lifespan extension is explained by the dependence on the sensitivity of the respective model to DIO when fed ad libitum. This could also explain contrasting results in primates where DR has no beneficial effect on age-related pathologies when the control group is already fed a limited amount of food to prevent hyperphagia [4] and hence possibly not subjected to accelerated cellular aging.

In this context, we have questioned whether DR applied as part of a ‘pro-aging’ energy dense diet would be able to reduce cellular aging in DIO-prone mice. We observed that hyperactivation of the mTOR pathway was abolished and that chaperone-mediated autophagy, known to decline with age, was induced compared with AL mice, suggesting that cellular rejuvenation is promoted in response to DR.

Using nutritional geometry approaches, the impact of dietary macronutrient composition on lifespan and hepatic mTOR activity was investigated. Interestingly, the diets that best support longevity were not the ones that best sustained reproductive success [1, 5]. It appears that extension of lifespan is achieved at the expense of reproduction and growth, and DR-related longevity may therefore be due to postponement of sexual reproduction.

An interesting target to investigate the interplay among nutrition, metabolism and reproductive behavior may be a family of small lipocalins called MUPs (major urinary proteins). MUPs are traditionally known as rodent urinary scent marks, but their involvement in energy and glucose metabolism points to an extended scope of biological effects. MUP expression is under control of steroid signaling that has been linked to nutrient-sensing pathways, including mTOR. Hepatic and urinary MUP levels are decreased upon DR treatment [6], including restriction of the energy dense ‘pro-aging’ diet [7]. Our data suggest that MUPs may be involved in the extension of murine lifespan through the inhibition of reproduction under conditions of dietary restriction. Furthermore, simple restriction of an energy dense ‘proaging’ diet exhibits similar health promoting effects, e.g., normalization of mTOR and insulin signaling, compared with what is considered a lifespan-extending dietaccording to Solon-Biet et al. [5]. In regards to the high-calorie, nutrient-dense diet consumed in Western societies and the subsequent health problems, it may be reasonable to suggest that restriction of the daily food amount would curb cellular senescence without the need to manipulate macronutrient composition.

## References

[1] Simpson SJ (2015). Cell.

[2] Blagosklonny MV (2006). Cell Cycle.

[3] Cuervo AM (2008). J Gerontol A Biol Sci Med Sci.

[4] Mattison JA (2012). Nature.

[5] Solon-Biet SM (2014). Cell Metabolism.

[6] Giller K (2013). Pro Biol Sci.

[7] Schloesser A (2015). Rejuvenation Research.

